# Expression of STAT3 and STAT6 in Men with Large Versus Small Prostates Undergoing Transurethral Resection of the Prostate

**DOI:** 10.3390/medicina62101990

**Published:** 2026-10-15

**Authors:** Chung Lyul Lee, Ji Yong Lee, Seung Woo Yang, Ju Hyun Shin, Ki Hak Song

**Affiliations:** 1 Department of Urology, College of Medicine, Chungnam National University, Daejeon 35015, Republic of Korea; mirusky92@naver.com (C.L.L.); sjh0402@cnu.ac.kr (J.H.S.); 2 U-Well Urologic Clinic, Daejeon 35233, Republic of Korea; urodragon@naver.com (J.Y.L.); white0941@hanmail.net (S.W.Y.)

**Keywords:** prostatic hyperplasia, STAT3 transcription factor, STAT6 transcription factor, lower urinary tract symptoms

## Abstract

*Background and Objectives*: The factors driving variations in prostate size in patients with benign prostatic hyperplasia (BPH) remain unclear. We evaluated the differential expression of signal transducer and activator of transcription (STAT) 3 and 6 in patients with BPH and large versus small prostates. *Materials and Methods*: Among men who underwent transurethral resection of the prostate (TURP), we defined, with reference to the mean prostate volume (PV) reported for Korean men aged 70 years or older, a small-prostate group (PV ≤ 33 mL) and a large-prostate group (PV > 60 mL). Basic demographics, serum prostate-specific antigen (PSA) levels, International Prostate Symptom Score (IPSS), quality of life (QoL), PV, and preoperative uroflowmetry values were evaluated. Tissue sections were assessed by immunofluorescence staining under a confocal laser-scanning microscope and histologically classified into prostatic gland and stroma. Mean fluorescence intensities of STAT3 and STAT6 were quantified using an image analyser. *Results:* STAT3 immunoreactivity was consistently higher in the stroma than in the glandular compartment in both groups (paired difference 43.1, *p* < 0.001), with no difference between volume groups in any compartment. Stromal STAT6 immunoreactivity was higher in the small-prostate group (65.2 ± 14.7 vs. 56.5 ± 14.1; mean difference 8.7, 95% CI 0.2–17.3, *p* = 0.045), and the estimate was similar in mixed-effects and generalised estimating equation models accounting for within-patient clustering (*p* = 0.030 and *p* = 0.030). This exploratory difference did not remain significant after correction for six comparisons (Holm-adjusted *p* = 0.269), and its correlations with clinical variables were not significant after correction. No difference was detected in the glandular or nodular compartments. *Conclusions:* STAT3 immunoreactivity showed a consistent stromal predominance across both volume groups, whereas stromal STAT6 immunoreactivity was higher in men with small prostates. This exploratory association was consistent across analytical approaches but did not survive correction for multiple comparisons. Because total rather than phosphorylated protein was measured, these data localise a candidate signal to the stromal compartment rather than demonstrating pathway activation. Given experimental evidence linking STAT6 signalling to prostatic fibrosis, the finding defines a testable hypothesis for phospho-specific and histological studies of stromal remodelling in men with small prostates.

## 1. Introduction

Benign prostatic hyperplasia (BPH) is a histologic observation characterised by the proliferation of epithelial and/or stromal cells in the periurethral area, leading to benign prostatic enlargement. The incidence of BPH increases significantly with age; approximately 50% of men aged 50–60 years, 70–80% of men aged 61–80 years, and 90% of men aged >80 years are affected [1,2,3]. Furthermore, the global burden of the disease has surged over the past 30 years, with the overall prevalence, incidence, and disability-adjusted life years increasing by 119–121% [4]. Accumulating evidence suggests that chronic prostatic inflammation significantly influences prostate enlargement and disease progression, and it has been hypothesised that prolonged exposure to inflammatory cytokines, tissue damage substrates, hypoxia, and continuous wound-healing results in BPH [5,6]. Robert et al. demonstrated that patients with chronic inflammation had higher prostate volume (PV), International Prostate Symptom Scores (IPSSs), and surgical intervention rates [7].

The Janus kinase/signal transducer and activator of transcription (JAK/STAT) pathway is a well-established inflammatory signalling cascade. This pathway regulates crucial biological processes, including cell proliferation, differentiation, apoptosis, immune regulation, and hematopoiesis [8]. The STAT family of proteins comprises seven subtypes; among them, STAT3 has been extensively studied in prostate cancer, demonstrating its oncogenic properties and robust effects on cellular proliferation [9]. In contrast, STAT6 is primarily stimulated during inflammation and functions as a T helper cell type 2 (Th2)-inducing transcriptional activator [10].

Although increasing evidence indicates that STATs induce prostate cancer cell proliferation [11], few studies have focused on their role in BPH. Investigating the factors that determine prostate size may improve our understanding of BPH progression and risk stratification. Therefore, this study aimed to elucidate whether STAT3 and STAT6 expression is associated with prostate enlargement in men with lower urinary tract symptoms (LUTS)/BPH.

## 2. Materials and Methods

### 2.1. Patient Population

Prostate tissues were obtained from patients with benign prostatic hyperplasia (BPH) who underwent transurethral resection of the prostate (TURP) at Chungnam National University Hospital between 2011 and 2018. Throughout this report we use BPH for the histological diagnosis, benign prostatic enlargement (BPE) for the size-based description, bladder outlet obstruction (BOO) for the urodynamic finding irrespective of cause, benign prostatic obstruction (BPO) for obstruction attributable to BPE, and lower urinary tract symptoms (LUTS) for the symptom complex. A nationwide screening study of Korean men reported a mean prostate volume of 34.4 mL in men aged 70 years or older [12]. Because the mean age of our cohort exceeded 70 years, we used this value as a reference and defined, by transrectal ultrasonography (TRUS)-estimated prostate volume (PV), a small-BPE group (PV ≤ 33 mL), at or below this reference, and a large-BPE group (PV > 60 mL), well above it. Individual ages ranged from 51 to 87 years, and a sensitivity analysis restricted to men aged ≥60 years was performed (see Section 2.3). Patients with prostate cancer on final pathology, neurogenic bladder, prior prostatic surgery, or insufficient archived tissue were excluded. Surgical indication was determined by comprehensive clinical judgement rather than by a predefined numerical threshold. Candidates were men with bothersome LUTS refractory to medical therapy, a low maximal flow rate on uroflowmetry, and a clinical impression that the prostate was the principal contributor to obstruction. Pressure-flow studies were performed selectively and at the discretion of the treating urologist, in an identical proportion of both groups (27/45, 60.0% of the large-BPE group and 9/15, 60.0% of the small-BPE group; *p* = 1.000), most often when flow rate and symptom burden were discordant with prostate size. Because patients were identified retrospectively from surgical and pathology records and prospective screening logs were not maintained over this period, the number of patients screened and excluded at each step could not be reconstructed, and no screening flow diagram is presented. Demographic data collected included age, body mass index (BMI), underlying diseases, and past medical history including urinary tract infection (UTI) and acute urinary retention. Preoperative medication exposure was recorded: no patient in either group was receiving a 5-alpha-reductase inhibitor or an anti-androgen at the time of surgery, all patients had received alpha-blocker therapy of variable and unrecorded duration, and use of aspirin and cyclo-oxygenase-2 inhibitors was recorded. Initial serum prostate-specific antigen (PSA) levels, International Prostate Symptom Score (IPSS), quality of life (QoL) scores, TRUS-guided PV, TRUS-measured intravesical prostatic protrusion (IPP) grade, preoperative uroflowmetry values, and pressure-flow parameters where available were evaluated. One small-BPE patient who was unable to void during uroflowmetry (maximal flow rate and voided volume of 0) was excluded from the uroflowmetry analyses. The Bladder Outlet Obstruction Index (BOOI) was calculated as PdetQmax − 2Qmax, with values > 40 defined as obstructed, 20–40 equivocal, and <20 unobstructed, according to the International Continence Society provisional nomogram. Intravesical prostatic protrusion (IPP) on transrectal ultrasonography was graded as grade 1 (≤5 mm), grade 2 (5–10 mm) or grade 3 (>10 mm).

### 2.2. Immunofluorescence (IF) Staining

One paraffin-embedded prostate tissue block was selected per patient and sectioned at a thickness of 4 μm onto saline-coated slides. For deparaffinisation and rehydration, slides were sequentially treated with xylene (20 min) and graded alcohols (100%, 95%, 90%, 80% and 70%; 10 min each), followed by distilled water and 0.01 M phosphate-buffered saline (PBS; pH 7.4). Antigen retrieval was performed by immersing slides in 1× sodium citrate buffer and microwaving for 5 min. To block non-specific binding, tissues were incubated with 10% normal goat serum (Abcam, Cambridge, UK) at room temperature for 1 hour. Slides were then incubated overnight at 4 °C with primary antibodies: anti-STAT6 rabbit monoclonal antibody (1:100; ab32520, Abcam) and anti-STAT3 mouse monoclonal antibody (1:100; ab119352, Abcam). After PBS washes, secondary antibodies—Alexa Fluor 488 goat anti-mouse (1:400; Invitrogen, Waltham, MA, USA) and Alexa Fluor 568 donkey anti-rabbit (1:400; Invitrogen)—were applied. 4′,6-diamidino-2-phenylindole (DAPI; 1:400; Sigma-Aldrich, St. Louis, MO, USA) was used for nuclear staining. Slides were washed with 0.01 M PBS containing 0.1% Triton X-100 and mounted using standard mounting medium (Dako, Santa Clara, CA, USA). All 60 specimens were stained in a single batch using a common antibody master mix, and were imaged in a single session on an LSM 880 confocal system with an AxioObserver stand (Carl Zeiss, Jena, Germany) using a Plan-Apochromat 20×/0.80 objective, at 1024 × 1024 pixels covering a 531.4 × 531.4 μm field (0.52 × 0.52 μm/pixel, 8-bit depth, 2-fold line averaging, 2.05 μs pixel dwell time). Channels were acquired line-sequentially to prevent spectral bleed-through: DAPI (405 nm excitation, 410–479 nm detection), Alexa Fluor 488 for STAT3 (488 nm, 491–535 nm) and Alexa Fluor 568 for STAT6 (594 nm, 595–674 nm). Acquisition settings were held constant across all specimens—laser transmission 0.86%, 0.96% and 0.75% for the 405, 488 and 594 nm lines respectively; photomultiplier detector gain 611.9, 883.6 and 989.5; detector offset 0 and digital gain 1.0 for all channels—so that acquisition conditions were identical for all patients. No post-acquisition adjustment of brightness or contrast was applied to the images used for quantification. Five non-overlapping fields were acquired per section, selected by the observer as histologically representative rather than by random sampling. Within each field, regions were segmented manually and classified as glandular (diffusely distributed acini), stromal (interacinar stroma) or nodular. A prostatic nodule was defined as a discrete, circumscribed hyperplastic lesion with a morphologically distinct border, corresponding to the transition zone hyperplastic nodules described by McNeal, which arise where prostatic glands and sphincteric stroma intermingle and are therefore mixed rather than purely glandular in composition [13,14]. A compartment not represented in a given field contributed no region, which accounts for the unequal denominators: 201 glandular regions from 59 patients (median 3 per patient, range 1–5), 173 stromal regions from 59 patients (median 3, range 1–5) and 54 nodular regions from 34 patients (median 1.5, range 1–3). Compartment assignment was reviewed and confirmed by a pathologist with genitourinary pathology expertise. All field selection, segmentation and quantification were performed blind to prostate-volume group assignment, and group allocation was linked to the quantitative data only after all measurements were complete. Signal was quantified using a ZEN 2.3 image analyser (Carl Zeiss Microscopy GmbH, Jena, Germany) as the mean fluorescence intensity (MFI) per unit area within each segmented region, expressed in 8-bit grey levels (0–255) and computed as the summed pixel intensity divided by region area. Because this is a mean intensity rather than an absorbance-based measurement, we use the term mean fluorescence intensity throughout. Intensities were not corrected for tissue autofluorescence; the implications are addressed in the Limitations. Commercially validated monoclonal antibodies were used for both targets: anti-STAT3 (ab119352) and anti-STAT6 (ab32520), both validated by the manufacturer. To assess antibody specificity qualitatively, dedicated negative and positive controls were subsequently performed and are presented as Supplementary Figure S2. Negative controls consisted of sections processed identically but with the primary antibodies omitted and replaced by an equal volume of antibody diluent; positive controls consisted of sections stained with the full protocol. Both controls were prepared for benign prostatic tissue from a patient not included in the study cohort and for prostate cancer tissue, the latter selected because STAT6 is reported to be selectively activated [15] and constitutively expressed [16], and STAT3 to be constitutively activated [9,11], in prostate carcinoma. All four control sections were stained in a single batch and imaged on an upright widefield epifluorescence microscope (DM2500; Leica Microsystems, Wetzlar, Germany) equipped with a DFC7000 T camera, with STAT3, STAT6 and DAPI acquired sequentially through the corresponding filter sets. The benign prostatic negative and positive controls were imaged on the same day at ×200 (HC PL FLUOTAR 20×/0.55 objective) with identical camera settings for each channel (STAT3 and STAT6: 300 ms exposure, gain 3; DAPI: 100 ms, gain 2). The prostate cancer negative and positive controls were imaged in the same session at ×200 on corresponding regions of serial sections from the same block. DAPI was acquired with identical settings; in the STAT3 and STAT6 channels the positive control was acquired with a modestly higher camera sensitivity (STAT3, 250 ms, gain 3; STAT6, 300 ms, gain 3) than the negative control (both 300 ms, gain 2). No post-acquisition adjustment of brightness or contrast was applied.

### 2.3. Statistical Analysis

The patient was the unit of analysis. Patient-level values were obtained by averaging the regions of interest scanned for each patient within each compartment, so that each patient contributes equally regardless of how many regions were available. Clinical continuous variables were compared using the independent-samples *t*-test, with Welch’s correction where Levene’s test indicated unequal variances, and the Bladder Outlet Obstruction Index using the Mann–Whitney U test; for marker intensities, Welch’s *t*-test and the Mann–Whitney U test are reported alongside the *t*-test. Categorical variables were assessed using the chi-square test, or the Fisher exact test when any expected count was below five. Effect estimates are presented as mean differences with 95% confidence intervals. Because the number of regions differed between patients, two sensitivity analyses retaining all regions were performed: a linear mixed-effects model with a random intercept for patient (restricted maximum likelihood), and generalised estimating equations with an exchangeable working correlation and patient as the cluster; intraclass correlation coefficients were derived from the mixed model. Estimates from these models are mean differences between groups that account for within-patient clustering only; no clinical covariates were included in any model. A sensitivity analysis restricted to men aged ≥60 years was performed. Associations between patient-level marker intensity and clinical variables were examined using Spearman rank correlation, with Holm adjustment across the clinical variables examined for each marker. No primary endpoint was designated a priori; all six compartment–protein comparisons were exploratory, and unadjusted *p* values are reported alongside Holm- and Benjamini–Hochberg-adjusted values. Analyses were performed using SPSS version 26.0 (IBM Corp., Armonk, NY, USA) and Python 3.13 (Python Software Foundation, Wilmington, DE, USA) with the statsmodels (version 0.15) and SciPy (version 1.18) packages. A *p* value < 0.05 was considered statistically significant.

### 2.4. Ethics Statement

This study was conducted in accordance with the Declaration of Helsinki. The protocol was approved by the Institutional Review Board of Chungnam National University Hospital (approval number: CNUH 201812044), and written informed consent was obtained from all patients.

## 3. Results

### 3.1. Clinical Characteristics

Baseline clinical characteristics, including age, BMI and medical history, showed no significant differences between the two groups. However, the acute urinary retention rate was significantly higher in the large-BPE group than in the small-BPE group (62.2% vs. 26.7%, *p* = 0.017; Table 1). The mean PVs were 25.3 ± 5.6 mL in the small-BPE group and 88.2 ± 31.6 mL in the large-BPE group (*p* < 0.001). Serum PSA levels were also significantly higher in the large-BPE group (8.69 ± 6.15 vs. 1.36 ± 1.66 ng/mL, *p* < 0.001). Preoperative anti-inflammatory medication use did not differ between groups (aspirin 40.0% vs. 20.0%, *p* = 0.169; cyclo-oxygenase-2 inhibitor 6.7% vs. 11.1%, *p* = 1.000). Urodynamic evaluation was performed in an identical proportion of each group (60.0%; *p* = 1.000). Among those studied, the Bladder Outlet Obstruction Index was substantially lower in the small-BPE group (39.3 ± 11.2 vs. 63.8 ± 28.3, *p* = 0.016), and only four of nine met the conventional obstructed threshold of >40, compared with 20 of 27 in the large-BPE group. TRUS-measured intravesical prostatic protrusion also differed markedly, with no patient in the small-BPE group showing grade 3 protrusion (0/15 vs. 25/45, *p* = 0.001).

The small-prostate group tended to show worse QoL scores (4.87 ± 0.92 vs. 4.29 ± 1.29, *p* = 0.067) and had larger voided volumes (253 ± 123 vs. 153 ± 90 mL, *p* = 0.003) than the large-prostate group. The IPSS and other uroflowmetry parameters, including maximal flow rate and post-void residual urine volume, did not differ significantly between the two groups, indicating that patients in both groups experienced LUTS and BPH despite the marked difference in prostate size (Table 2).

### 3.2. STAT3 and STAT6 Immunofluorescence (IF) Staining Under Confocal Laser-Scanning Microscopy

**Figure 1:**
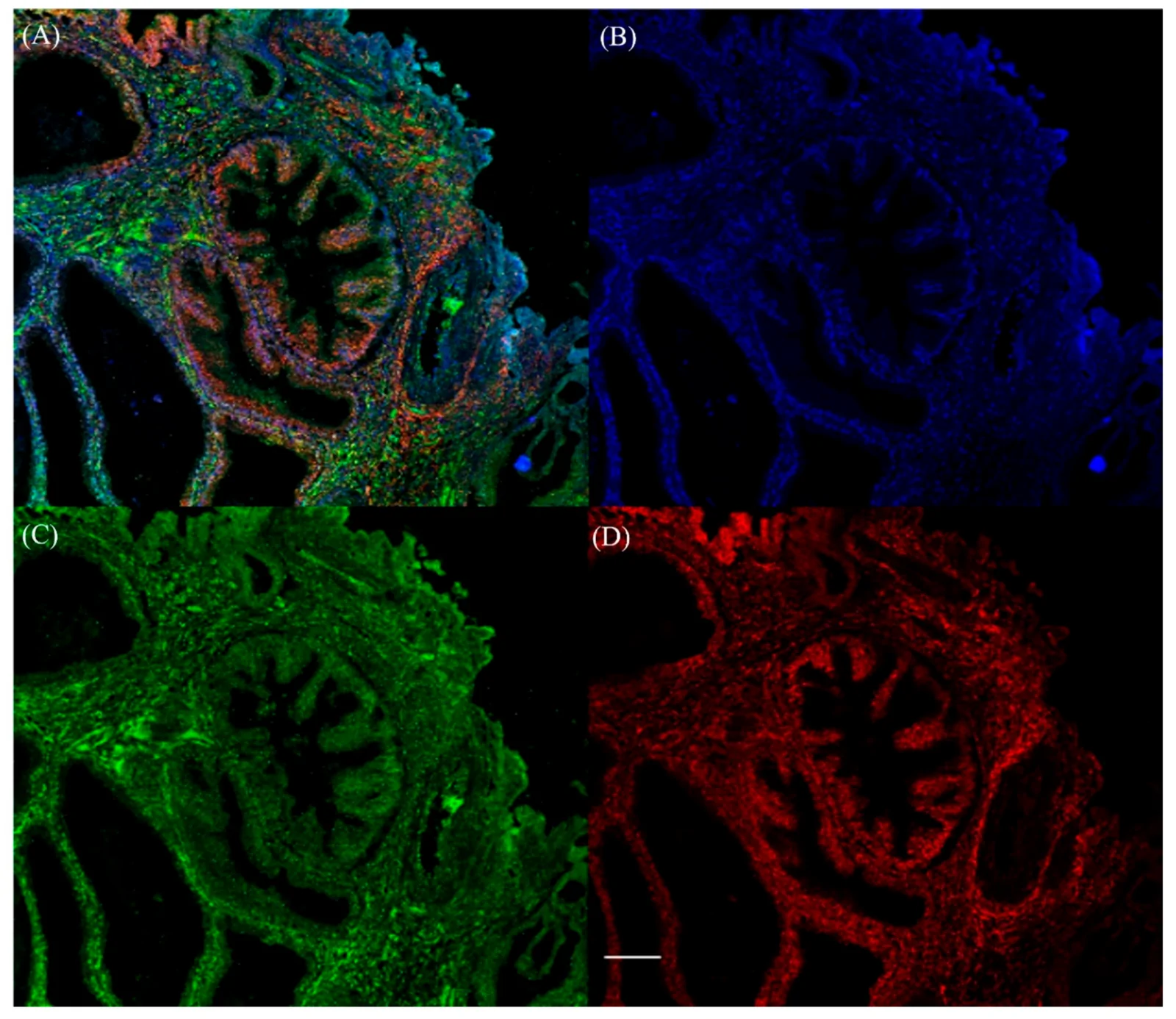
Colocalisation of STAT3 and STAT6 by immunofluorescence staining. Microphotographs of multiplex immunofluorescence using 4′,6-diamidino-2-phenylindole (DAPI) (blue) (**B**), signal transducer and activator of transcription (STAT) 3 (green) (**C**), and STAT6 (red) (**D**). The merged image is shown in (**A**). STAT3 expression was increased predominantly in the prostatic stroma, whereas STAT6 expression was similarly distributed between the prostatic gland and stroma. Images were acquired with a Plan-Apochromat 20×/0.80 objective. Scale bar = 50 μm.

Multiplex IF analysis revealed distinct compartment-specific expression patterns of STAT3 and STAT6 in the prostate. Confocal microscopy showed that STAT3 was strongly expressed in the interstitial spaces between the glands, particularly within the prostatic stroma, whereas STAT6 was similarly expressed in the prostatic glandular and stromal compartments (Figure 1).

The control sections provided qualitative support for staining specificity (Supplementary Figure S2). In benign prostatic tissue imaged with matched acquisition settings, the section processed without primary antibodies showed only minimal signal in the STAT3 and STAT6 channels despite preserved nuclear staining, whereas the section stained with the complete protocol showed distinct signal in both channels. In prostate cancer tissue, used as a positive tissue control, corresponding regions of serial sections showed distinct signal in both channels with the complete protocol and minimal signal without primary antibodies; because the positive section was acquired with a higher camera sensitivity, this comparison is presented qualitatively only. Faint punctate signal present irrespective of primary antibody was attributable to tissue autofluorescence. Matched acquisition settings do not exclude section-to-section differences in background fluorescence, and the controls were therefore not used for quantitative comparison.

**Figure 2:**
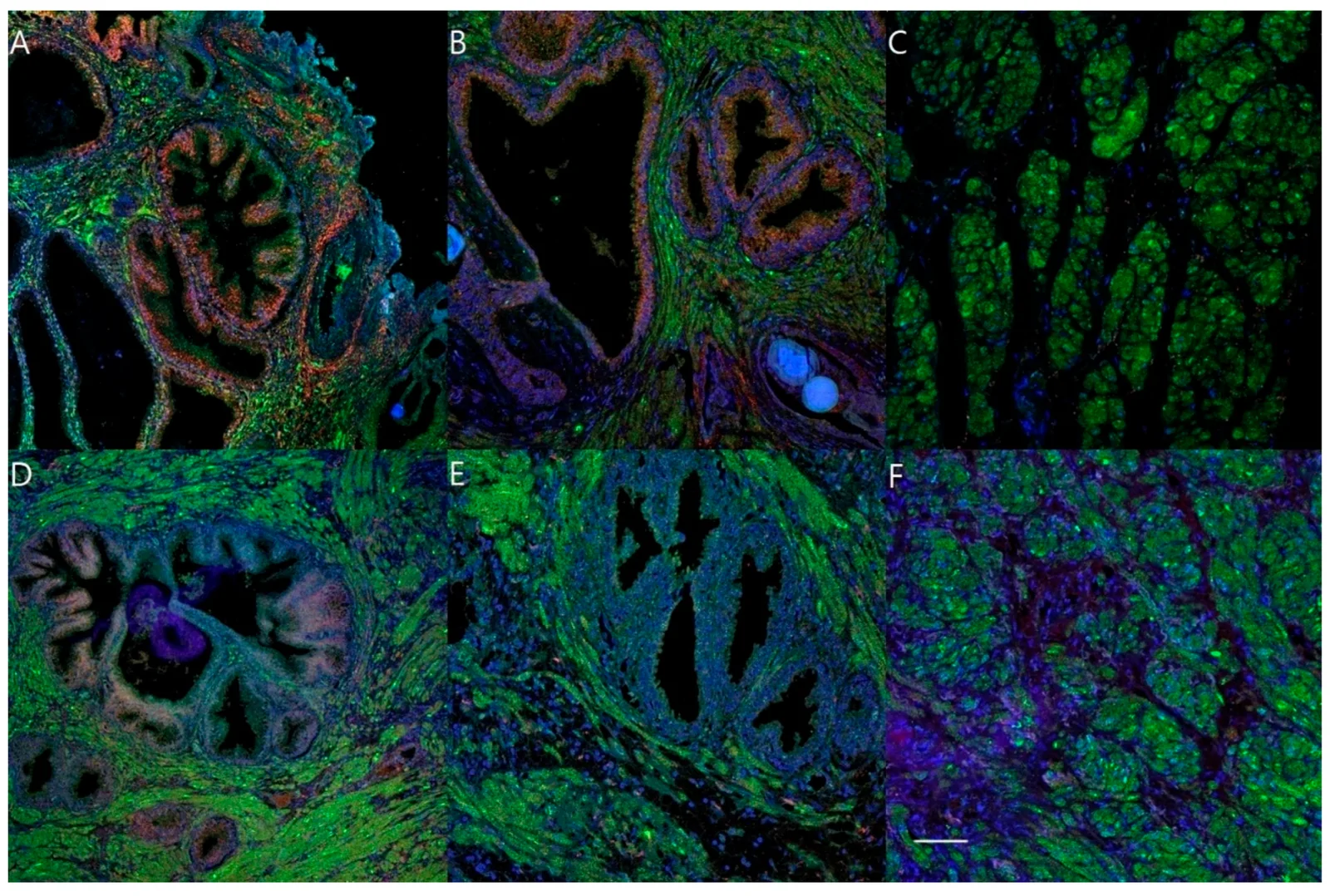
Colocalisation of STAT3 and STAT6 by immunofluorescence staining. Microphotographs (merged images) of representative examples of multiplex immunofluorescence using DAPI (blue), STAT3 (green) and STAT6 (red). Panels (**A**–**C**) show the large-BPE group and panels (**D**–**F**) the small-BPE group: large-BPE group showing STAT6 expression of the prostatic gland and STAT3 of the stroma (**A**,**B**) and STAT3 expression of the prostatic nodule (**C**); small-BPE group showing STAT6 expression of the prostatic gland and STAT3 of the stroma (**D**,**E**) and STAT3 expression of the prostatic nodule (**F**). STAT3 expression was stronger in the stromal and nodular compartments than in the glandular compartment in both groups. Stromal STAT6 expression was stronger in the small-BPE group. Images were acquired with a Plan-Apochromat 20×/0.80 objective. Scale bar = 50 μm.

Quantitative analysis at the patient level demonstrated that STAT3 immunoreactivity was consistently higher in the stromal than in the glandular compartment in both groups (paired difference 43.1, *p* < 0.001; Figure 2). No significant difference was observed between the volume groups in any compartment (large-BPE vs. small-BPE): glandular, 75.8 ± 14.2 vs. 75.9 ± 15.1 (difference 0.07, 95% CI −8.74 to 8.88, *p* = 0.987); stromal, 119.6 ± 29.6 vs. 127.8 ± 44.3 (difference 8.25, 95% CI −12.00 to 28.49, *p* = 0.418); and nodular, 139.8 ± 45.7 vs. 135.1 ± 32.3 (difference −4.70, 95% CI −35.08 to 25.67, *p* = 0.754). Nodular STAT3 intensity exceeded both glandular (paired difference 59.9, *p* < 0.001) and stromal intensity (paired difference 13.5, *p* = 0.032), consistent with the mixed stromal–glandular composition of transition zone nodules.

In contrast, stromal STAT6 immunoreactivity was higher in the small-BPE group than in the large-BPE group (65.2 ± 14.7 vs. 56.5 ± 14.1; difference 8.74, 95% CI 0.21 to 17.28, *p* = 0.045; Figure 2 and Table 3). Welch’s *t*-test (*p* = 0.056) and the Mann–Whitney U test (*p* = 0.061) placed this difference at the margin of conventional significance. Sensitivity analyses retaining all regions and accounting for within-patient clustering gave concordant estimates: linear mixed-effects model, mean difference 9.51 (95% CI 0.92 to 18.11, *p* = 0.030); generalised estimating equations, 9.48 (95% CI 0.93 to 18.03, *p* = 0.030). In a sensitivity analysis restricted to men aged ≥60 years (14 small-BPE and 43 large-BPE), the estimate was similar (8.87, 95% CI 0.15 to 17.58, *p* = 0.046; Supplementary Table S3). Intraclass correlation coefficients ranged from 0.22 to 0.65 across compartments, confirming that regions from the same patient were not independent. No significant difference was observed in the glandular compartment (59.8 ± 20.3 vs. 54.8 ± 19.7, difference 5.00, 95% CI −7.16 to 17.15, *p* = 0.414) or the nodular compartment (58.2 ± 13.7 vs. 59.6 ± 20.3, difference −1.43, 95% CI −14.82 to 11.97, *p* = 0.829). Because all six compartment–protein comparisons were exploratory, the stromal STAT6 result should be interpreted in light of multiplicity: it did not remain significant after Holm correction (adjusted *p* = 0.269) or Benjamini–Hochberg control of the false discovery rate (q = 0.269). Post hoc power for this comparison was 52% (Cohen’s d = 0.61). At the patient level, no correlation between stromal STAT6 and the clinical variables examined was significant after Holm correction (Supplementary Table S2): IPSS (rho = −0.145, *p* = 0.273), QoL (rho = −0.061, *p* = 0.644), post-void residual volume (rho = 0.005, *p* = 0.971), prostate volume (rho = −0.068, *p* = 0.609) and the Bladder Outlet Obstruction Index (rho = −0.164, *p* = 0.348). A nominal association was observed with maximal flow rate (rho = 0.359, *p* = 0.007; Holm-adjusted *p* = 0.078), which did not remain significant after correction.

## 4. Discussion

Managing BPH remains one of the most critical issues in men’s urological health, as LUTS and their associated complications profoundly affect patient QoL. BPH pathogenesis is driven by a complex, interconnected web of factors, including androgens, stromal–epithelial interactions, apoptosis regulation, inflammatory pathways, growth factors, and neurotransmitters. Despite extensive research efforts to map these pathways, the definitive cause-and-effect relationships governing prostate growth remain unclear.

We defined the groups with reference to the mean PV of 34.4 mL reported for Korean men aged 70 years or older in a nationwide screening population [12]; because the mean age of both groups exceeded 70 years, this age-matched value was a reasonable reference for separating small from large prostates. The small-BPE group (≤33 mL) lay at or below this reference and the large-BPE group (>60 mL) well above it, an extreme-group design intended to maximise the contrast in prostate size. Surgery in the small-BPE cohort was undertaken on the basis of comprehensive clinical judgement, and the accrual of only 15 such patients over eight years reflects a clinical reality rather than incomplete recruitment: in Korean tertiary practice, men presenting with LUTS and a small prostate are uncommon, and among those who do present surgery is rarely the chosen management.

We acknowledge that current guidelines generally reserve transurethral resection of the prostate for prostates of approximately 30–80 mL and recommend transurethral incision of the prostate as an option for glands ≤30 mL without an obstructive middle lobe. In the small-BPE group, resection rather than incision was chosen because cystoscopy demonstrated lateral and, in some cases, median lobe enlargement considered to require resection, and because pressure-flow findings in these patients were consistent with obstruction; the need to obtain sufficient tissue for compartment-specific analysis was a secondary consideration. This selection does not affect the internal validity of the tissue comparison, but it does limit the generalisability of the small-prostate surgical cohort, since such patients would more commonly be managed by incision or by non-surgical means.

STAT3 is a well-known oncogenic factor in many malignancies and actively promotes prostate cancer cell proliferation [9]. During BPH progression, the JAK2/STAT3 pathway stimulates cytokines and growth factors that drive cell proliferation, survival and angiogenesis [17]. In benign prostatic tissue specifically, Ou et al. showed that mast cells infiltrating human BPH tissue release interleukin-6, which activates STAT3/Cyclin D1 signalling in BPH-1 cells and promotes their proliferation; blockade of either interleukin-6 or STAT3 partially abrogated this effect [18]. Initially, we hypothesised that STAT3 expression would be higher in patients with large prostates. However, STAT3 was more strongly expressed in the stroma than in the glandular compartment in both groups and was relatively consistent regardless of PV. Set against the experimental evidence above, proliferation and prostatic growth are not simply coupled in established BPH: using Ki-67, Claus et al. found no significant correlation between proliferation rate and age in either the epithelium or stroma of hyperplastic prostates, nor between prostate weight and proliferation rate, although proliferation in BPH tissue greatly exceeded that in normal prostate—ninefold in epithelium and thirty-sevenfold in stroma [19]. Their specimens were obtained by suprapubic prostatectomy and were uniformly large, so their findings do not extend to small prostates; the disproportionately stromal component of that increase nonetheless supports a compartment-specific approach. Taken together, these observations indicate that STAT3 is proliferation-competent in benign prostatic cells, yet neither STAT3 abundance nor Ki-67-defined proliferation appears to track prostate size in established disease. We emphasise that we measured total rather than phosphorylated STAT3, so a difference in pathway activation between groups can be neither demonstrated nor excluded by our data.

In the REDUCE trial, histological evidence of chronic inflammation was observed in 77.6% of patients with LUTS/BPH [20]. Chronic inflammation triggers persistent lymphocyte infiltration, cytokine release and localised hypoxia, initiating a continuous wound-healing cascade modulated by multiple growth factors and driving simultaneous stromal and epithelial proliferation [6] and, when sustained, extracellular matrix deposition. Inflammation was not assessed in the present cohort: inflammatory infiltration was not graded and inflammatory cell markers were not included in the immunofluorescence panel, so an unmeasured difference in inflammatory burden between the groups remains a plausible alternative explanation for the difference we observed in stromal STAT6.

Kang et al. reported that men with small prostates and LUTS had symptom scores and uroflowmetry indices comparable to those of men with larger prostates and demonstrable BOO, despite less-frequent urodynamic obstruction [21]. Our data show the same pattern. IPSS (23.3 ± 5.5 vs. 22.5 ± 6.7, *p* = 0.695) and maximal flow rate (9.0 ± 3.7 vs. 8.0 ± 4.7 mL/s, *p* = 0.535) did not differ between our small-BPE and large-BPE groups despite a more than threefold difference in prostate volume, and among patients who underwent pressure-flow studies, the Bladder Outlet Obstruction Index was substantially lower in the small-BPE group (39.3 ± 11.2 vs. 63.8 ± 28.3, *p* = 0.016), with only four of nine meeting the conventional obstructed threshold compared with 20 of 27. The correspondence between the two studies is therefore at the level of this clinical observation—that symptom burden in men with small prostates is disproportionate to both gland size and the degree of demonstrable obstruction—and not at the level of mechanism.

Although benign prostatic obstruction due to BPE is the most common cause of BOO in older men, many men experience LUTS and obstruction without marked prostatic enlargement [22]. Prostatic fibrosis has been proposed as one non-volumetric contributor: periurethral prostatic tissue from men with LUTS is stiffer and contains more collagen than that from men without LUTS [23], and an increased periurethral extracellular matrix may reduce urethral compliance and raise outlet resistance independently of gland size [24].

Several lines of evidence link STAT6 to this process. In the human prostate, STAT6 is expressed prominently in the fibromuscular stroma [16]. In BPH, interleukin-4 derived from M2 macrophages induces fibroblast-to-myofibroblast transition and collagen deposition through JAK/STAT6, together with PI3K/AKT and MAPK/ERK signalling [25], and interleukin-4 and interleukin-13 receptors are co-upregulated with collagen in periurethral tissue from men with LUTS, where this signalling axis drives collagen production by prostatic fibroblasts [26]. Outside the prostate, STAT6-deficient mice develop less tubulointerstitial fibrosis after ureteral obstruction [27]. Against this background, the higher stromal STAT6 immunoreactivity in men with small prostates is an observation that may be relevant. In our cohort, symptom severity and maximal flow rate in the small-BPE group were comparable to those in the large-BPE group despite the large difference in prostate volume, and the mean Bladder Outlet Obstruction Index in the small-BPE group (39.3) lay close to the obstructed threshold even though these glands averaged only 25 mL. We therefore hypothesise that STAT6-associated stromal remodelling contributes to outlet resistance, or to reduced urethral compliance, that is disproportionate to gland size in men with small prostates who come to TURP. This hypothesis is not tested by the present data. Fibrosis, collagen content, myofibroblast markers and phosphorylated STAT6 were not measured, and the only nominal association between stromal STAT6 and a flow measure—a weak positive correlation with maximal flow rate (rho = 0.359) that did not survive correction—runs opposite to the direction the hypothesis predicts. The hypothesis does, however, specify measurements for future work: quantification of collagen (for example, by picrosirius red staining), α-smooth muscle actin and phosphorylated STAT6 within the same stromal compartment, correlated with pressure–flow parameters in men across the range of prostate volumes.

### 4.1. Strengths and Limitations

This study provides compartment-specific data on STAT3 and STAT6 expression in surgically treated men with small and large prostates, a comparison that has received little attention. All specimens were stained in a single batch and imaged under fixed acquisition settings, field selection and quantification were blinded to group, the patient was the unit of analysis, and the stromal STAT6 difference was consistent across analyses accounting for within-patient clustering and in a sensitivity analysis restricted to men aged ≥60 years. These features should be weighed against the limitations below.

#### 4.1.1. Design and Population

The cohort was modest and unequal in size (45 versus 15), and post hoc power for the stromal STAT6 comparison was 52%; the confidence interval for the group difference (0.21 to 17.28) is correspondingly wide and includes values of negligible biological importance. We compared prostates of 33 mL or less with those above 60 mL and excluded the intermediate range in which most men with BPE fall; this extreme-group design increases contrast but prevents assessment of a dose–response relationship and inflates the effect estimate relative to an unselected cohort. All patients underwent TURP, so both groups represent men whose symptoms warranted surgery, and neither is representative of community-dwelling men with LUTS. Surgical indication rested on clinical judgement rather than a predefined threshold, and urodynamic evaluation was performed in 60% of each group at the discretion of the treating urologist rather than by protocol, so the urodynamic data are subject to indication bias and are presented descriptively. Individual ages ranged from 51 to 87 years; a sensitivity analysis restricted to men aged ≥60 years gave consistent results (Supplementary Table S3).

A history of acute urinary retention was significantly more frequent in the large-BPE group (62.2% versus 26.7%). Retention is associated with prostatic ischaemia, inflammatory infiltration and subsequent tissue remodelling, and is therefore a plausible confounder of stromal protein expression rather than a neutral baseline characteristic; this study cannot separate the effect of volume group from that of retention history. A history of UTI was also numerically more frequent in the large-BPE group (28.9% versus 6.7%), and men with retention would have been catheterised, both of which induce local inflammation. No patient was receiving a 5-alpha-reductase inhibitor at the time of surgery, but remote exposure at referring institutions could not be ascertained; alpha-blocker exposure was universal and therefore cannot account for between-group differences, but its duration was variable and not recorded systematically. Screening and exclusion counts could not be reconstructed from records spanning 2011–2018, which limits assessment of selection bias.

#### 4.1.2. Tissue Sampling and Measurement

TURP chips derive from the transition zone and periurethral tissue and do not represent the peripheral zone or the prostate as a whole. Because the transition zone is the site of nodule formation in BPH, this sampling is appropriate to the question, but the findings cannot be generalised to whole-gland expression, and chip fragmentation and cautery artefact may affect quantification at fragment margins. One block and one section were examined per patient, and fields were selected by the observer as histologically representative rather than by random sampling; this introduces a potential selection effect, mitigated but not eliminated by blinding to group allocation.

Measured intensities were not corrected for tissue autofluorescence. This is of particular relevance to the STAT3 channel, whose detection window (491–535 nm) overlaps the emission of collagen and elastin autofluorescence under 488 nm excitation; part of the apparent stromal predominance of STAT3 may therefore be attributable to matrix autofluorescence rather than to specific antibody binding. The STAT6 channel (595–674 nm) lies in a spectral region where tissue autofluorescence is substantially lower. Single-batch staining and identical acquisition settings reduce, but do not exclude, differences in background fluorescence between sections, and uncorrected autofluorescence limits interpretation of the absolute compartmental difference in STAT3. Nodular regions were identified by morphological appearance rather than by a pre-specified classification scheme, and nodular subtypes were not distinguished; identifiable nodules were present in only 34 of 60 patients, so the nodular comparisons are the least well powered in the study. Finally, we measured total STAT3 and STAT6 protein, which reflects abundance rather than pathway activation; phosphorylated STAT proteins and nuclear translocation were not assessed.

Negative and positive controls in benign prostatic and prostate cancer tissue (Supplementary Figure S2) provide qualitative support for antibody specificity but do not establish it quantitatively. Only the benign prostatic controls were acquired with matched camera settings; the prostate cancer positive control was acquired with a modestly higher camera sensitivity in the STAT3 and STAT6 channels than its negative control. Isotype controls were not performed, and the controls were acquired on a widefield epifluorescence microscope in a separate imaging session, rather than on the confocal system used for the main analysis, so control and study images can be compared only qualitatively. Punctate autofluorescent signal was observed in both channels of the negative controls and is attributable to lipofuscin and corpora amylacea rather than to specific antibody binding; matched staining and acquisition conditions do not exclude section-to-section differences in such background signal, which may add variability to the between-group comparison.

#### 4.1.3. Analysis

No primary endpoint was designated a priori, and all six compartment–protein comparisons were exploratory. The stromal STAT6 difference did not remain significant after Holm correction or control of the false discovery rate. Because the number of regions analysed differed between patients, the patient-level analysis weights each patient equally regardless of how many regions contributed; sensitivity analyses retaining all regions and accounting for within-patient clustering gave concordant estimates. No correlation between patient-level stromal STAT6 and the clinical parameters examined was significant after correction for multiple comparisons. This is consistent with the restricted range of symptom severity in a surgical cohort (IPSS 10–35, with 63% scoring ≥ 20; Qmax 0.8–21 mL/s, with 95% below 15 mL/s) and with the absence of any between-group difference in IPSS or maximal flow rate, but it means that the tissue-level difference we report has no demonstrated individual-level clinical correlate in this cohort.

#### 4.1.4. Questions This Study Does Not Address

Histological inflammation and fibrosis were not quantified, so the inflammatory and fibrotic interpretations discussed above are drawn from the literature rather than from measurements in this cohort, and the relationship between STAT3 expression and proliferative activity was not examined. The cohort included no men younger than 50 years and no longitudinal measurements, so the study cannot address whether STAT expression differs in early-onset BPH or changes with prostate growth. Finally, the design is cross-sectional and observational, and expression was measured at a single time point late in the disease course; no causal or temporal inference is possible.

## 5. Conclusions

STAT3 immunoreactivity showed a consistent stromal predominance across both volume groups, whereas stromal STAT6 immunoreactivity was higher in men with small prostates (mean difference 8.7; 95% CI 0.2–17.3). This association was consistent across analyses accounting for within-patient clustering, but it was exploratory, did not remain significant after correction for multiple comparisons, and was not accompanied by any clinical correlation that survived correction. Because total rather than phosphorylated protein was measured, these data localise a candidate signal to the stromal compartment rather than demonstrating pathway activation. Together with experimental evidence linking STAT6 signalling to prostatic fibrosis, the finding defines a testable hypothesis—that STAT6-associated stromal remodelling contributes to symptoms disproportionate to gland size in men with small prostates—for phospho-specific, transcriptomic and histological studies.

## Figures and Tables

**Table 1:** Demographics, medication exposure and urodynamic findings of the study population within the large-BPE group (>60 mL) and small-BPE group (≤33 mL).

	Large-Prostate Group (n = 45)	Small-Prostate Group (n = 15)	*p* Value
Age (yr)	72.31 ± 7.03	73.87 ± 8.77	0.489
BMI (kg/m^2^)	24.42 ± 2.95	24.16 ± 3.36	0.776
Diabetes mellitus	13 (28.9)	3 (20.0)	0.738
Hypertension	17 (37.8)	8 (53.3)	0.290
UTI history	13 (28.9)	1 (6.7)	0.155
Acute urinary retention history	28 (62.2)	4 (26.7)	0.017
Prostate volume (mL)	88.24 ± 31.55	25.33 ± 5.60	<0.001
Transitional zone volume (mL)	45.77 ± 25.29	7.60 ± 3.44	<0.001
Longitudinal length (mm)	62.07 ± 7.74	40.95 ± 4.76	<0.001
PSA (ng/mL)	8.69 ± 6.15	1.36 ± 1.66	<0.001
Alpha-blocker use	45 (100)	15 (100)	–
5-ARI at time of surgery	0 (0)	0 (0)	–
Aspirin use	9 (20.0)	6 (40.0)	0.169
COX-2 inhibitor use	5 (11.1)	1 (6.7)	1.000
Urodynamic study performed	27 (60.0)	9 (60.0)	1.000
BOOI, if performed	63.8 ± 28.3	39.3 ± 11.2	0.016
BOOI > 40	20/27 (74.1)	4/9 (44.4)	0.126
TRUS-IPP grade 3	25 (55.6)	0 (0)	0.001

Values are presented as mean ± standard deviation or number (%). Continuous variables were analysed using the independent *t*-test, except the Bladder Outlet Obstruction Index (BOOI), which was compared using the Mann–Whitney U test. Categorical variables were analysed using the chi-square or Fisher exact test. Urodynamic parameters are reported only for the 27 large-BPE and nine small-BPE patients who underwent pressure-flow studies. BMI, body mass index; BOOI, Bladder Outlet Obstruction Index; BPE, benign prostatic enlargement; COX-2, cyclo-oxygenase-2; IPP, intravesical prostatic protrusion; PSA, prostate-specific antigen; TRUS, transrectal ultrasonography; UTI, urinary tract infection; 5-ARI, 5-alpha-reductase inhibitor.

**Table 2:** Voiding questionnaires and uroflowmetry findings in the large-BPE and small-BPE groups.

	Large-Prostate Group (n = 45)	Small-Prostate Group (n = 15)	*p* Value
IPSS	22.51 ± 6.70	23.27 ± 5.50	0.695
Storage symptom score	9.04 ± 3.10	8.53 ± 2.70	0.571
Voiding symptom score	13.47 ± 4.55	14.73 ± 4.32	0.348
QoL score	4.29 ± 1.29	4.87 ± 0.92	0.067
Maximal flow rate (mL/s)	8.05 ± 4.67	8.96 ± 3.70	0.535
Voiding volume (mL)	153 ± 90	253 ± 123	0.003
Residual urine volume (mL)	95 ± 75	90 ± 88	0.860

Values are presented as mean ± standard deviation. Continuous variables were analysed using the independent *t*-test, with Welch’s correction for QoL score, for which Levene’s test indicated unequal variances. Uroflowmetry was available in 44 patients in the large-BPE group and 12 patients in the small-BPE group; one small-BPE patient who was unable to void was excluded. BPE, benign prostatic enlargement; IPSS, International Prostate Symptom Score; QoL, quality of life.

**Table 3:** Patient-level comparison of STAT3 and STAT6 mean fluorescence intensity between the small-BPE and large-BPE groups.

		Large-BPE Group (n = 45/22/44)	Small-BPE Group (n = 14/12/15)	Difference (Small–Large), 95% CI	*p* Value
Prostate gland	STAT3	75.8 ± 14.2	75.9 ± 15.1	0.07 (−8.74 to 8.88)	0.987
	STAT6	54.8 ± 19.7	59.8 ± 20.3	5.00 (−7.16 to 17.15)	0.414
Prostate nodule	STAT3	139.8 ± 45.7	135.1 ± 32.3	−4.70 (−35.08 to 25.67)	0.754
	STAT6	59.6 ± 20.3	58.2 ± 13.7	−1.43 (−14.82 to 11.97)	0.829
Prostate stroma	STAT3	119.6 ± 29.6	127.8 ± 44.3	8.25 (−12.00 to 28.49)	0.418
	STAT6	56.5 ± 14.1	65.2 ± 14.7	8.74 (0.21 to 17.28)	0.045

Values are presented as mean ± standard deviation of the patient-level mean fluorescence intensity, with the patient as the unit of analysis. Differences are small-BPE minus large-BPE, compared using the independent-samples *t*-test; *p* values are unadjusted. Across the six exploratory comparisons, the Holm-adjusted *p* value was 0.269 for stromal STAT6 and 1.000 for all other comparisons. Denominators differ by compartment because not every compartment was represented in every patient’s scanned fields (glandular n = 45/14, nodular n = 22/12, stromal n = 44/15 for large/small groups respectively). Sensitivity analyses retaining all regions of interest are presented in Supplementary Table S1. STAT, signal transducer and activator of transcription; BPE, benign prostatic enlargement; CI, confidence interval.

## Data Availability

The raw data supporting the conclusions of this article will be made available by the authors on reasonable request, subject to institutional ethical approval.

## Supplementary Materials

The following supporting information can be downloaded at: https://www.mdpi.com/article/10.3390/medicina62101990/s1, Table S1: Clustering-aware sensitivity analyses of STAT3 and STAT6 mean fluorescence intensity between the small-BPE and large-BPE groups; Table S2: Patient-level Spearman correlations between stromal marker intensity and clinical variables; Table S3: Sensitivity analysis restricted to men aged ≥60 years: patient-level STAT3 and STAT6 mean fluorescence intensity between the small-BPE and large-BPE groups; Figure S1: Patient-level distribution of STAT3 and STAT6 mean fluorescence intensity by prostate-volume group; Figure S2: Negative and positive immunofluorescence controls.

## Abbreviations

The following abbreviations are used in this manuscript:

BMI	body mass index
BOO	bladder outlet obstruction
BPH	benign prostatic hyperplasia
DAPI	4′,6-diamidino-2-phenylindole
IF	immunofluorescence
IPSS	International Prostate Symptom Score
JAK	Janus kinase
LUTS	lower urinary tract symptoms
PBS	phosphate-buffered saline
PSA	prostate-specific antigen
PV	prostate volume
QoL	quality of life
STAT	signal transducer and activator of transcription
Th2	T helper cell type 2
TRUS	transrectal ultrasonography
TURP	transurethral resection of the prostate
UTI	urinary tract infection
BOOI	Bladder Outlet Obstruction Index
BPE	benign prostatic enlargement
BPO	benign prostatic obstruction
CI	confidence interval
GEE	generalised estimating equations
ICC	intraclass correlation coefficient
IPP	intravesical prostatic protrusion
MFI	mean fluorescence intensity
ROI	region of interest
5-ARI	5-alpha-reductase inhibitor
