# Supplementary material for: Expression of STAT3 and STAT6 in Men with Large Versus Small Prostates Undergoing Transurethral Resection of the Prostate
Source: Medicina (Kaunas). 2026 Oct 15;62(10):1990. doi: 10.3390/medicina62101990 (PMC13706706; doi:10.3390/medicina62101990)
Supplement: Supplementary file 1 [file medicina-62-01990-s001.zip › medicina-4622945-supplementary.pdf]

Supplementary Materials

Expression of STAT3 and STAT6 in Men with Large versus Small Prostates Undergoing Transurethral Resection of the Prostate

Chung Lyul Lee, Ji Yong Lee, Seung Woo Yang, Ju Hyun Shin, Ki Hak Song

Table S1. Clustering-aware sensitivity analyses of STAT3 and STAT6 mean fluorescence intensity between the small-BPE and large-BPE groups.

Compartment	Protein	Mixed model β (95% CI)	p	GEE β (95% CI)	p	ICC
Gland	STAT3	0.11 (−8.26 to 8.48)	0.980	0.11 (−8.29 to 8.51)	0.980	0.36
Gland	STAT6	5.61 (−6.49 to 17.71)	0.363	5.51 (−6.02 to 17.05)	0.349	0.65
Nodule	STAT3	−3.52 (−30.04 to 23.01)	0.795	−3.28 (−26.85 to 20.28)	0.785	0.22
Nodule	STAT6	−1.07 (−13.36 to 11.23)	0.865	−0.80 (−11.79 to 10.19)	0.886	0.60
Stroma	STAT3	4.58 (−14.52 to 23.67)	0.638	3.72 (−16.18 to 23.61)	0.714	0.43
Stroma	STAT6	9.51 (0.92 to 18.11)	0.030	9.48 (0.93 to 18.03)	0.030	0.57

β is the model-estimated mean difference (small-BPE minus large-BPE) in mean fluorescence intensity, accounting for within-patient clustering only; no clinical covariates were included. The mixed model is a linear mixed-effects model with a random intercept for patient, fitted by restricted maximum likelihood. GEE, generalised estimating equations with an exchangeable working correlation and the patient as the cluster; CI, confidence interval; ICC, intraclass correlation coefficient.

The corresponding patient-level analysis reported in Table 3 of the main text gave a difference of 8.74 (95% CI 0.21 to 17.28, $p = 0.045$) for stromal STAT6; Welch's t test gave $p = 0.056$ and the Mann–Whitney U test $p = 0.061$. Post-hoc power at the patient level was 52% (Cohen's $d = 0.61$); approximately 29 small-BPE and 84 large-BPE patients would be required for 80% power at the observed effect size. This comparison was one of six exploratory compartment–protein comparisons and did not remain significant after Holm correction (adjusted p = 0.269) or Benjamini–Hochberg control of the false discovery rate (q = 0.269).

Table S2. Patient-level Spearman correlations between stromal marker intensity and clinical variables.

Clinical variable	n	STAT6 rho	p	Holm p	STAT3 rho	p	Holm p
IPSS, total	59	−0.145	0.273	1.000	0.198	0.133	1.000
IPSS, storage subscore	59	−0.194	0.141	1.000	0.043	0.746	1.000
IPSS, voiding subscore	59	−0.113	0.395	1.000	0.261	0.046	0.446
QoL score	59	−0.061	0.644	1.000	0.059	0.656	1.000
Maximal flow rate	55	0.359	0.007	0.078	0.188	0.170	1.000
Voided volume	55	0.229	0.093	0.934	0.275	0.043	0.446
Post-void residual volume	54	0.005	0.971	1.000	−0.280	0.041	0.446
Prostate volume	59	−0.068	0.609	1.000	−0.090	0.499	1.000

Transition zone volume	59	−0.117	0.379	1.000	−0.127	0.339	1.000
BOOI (A/G number)	35	−0.164	0.348	1.000	0.092	0.601	1.000
Serum PSA	59	−0.199	0.131	1.000	−0.176	0.181	1.000

Holm-adjusted *p* values are computed separately within each marker across the eleven comparisons. BOOI values are available only for the patients who underwent pressure-flow studies. IPSS, International Prostate Symptom Score; QoL, quality of life; BOOI, Bladder Outlet Obstruction Index; PSA, prostate-specific antigen.

Four of the twenty-two correlations reached nominal significance and none survived Holm correction. Stromal STAT6 correlated weakly with maximal flow rate (rho = 0.359, p = 0.007, adjusted p = 0.078); the association with maximal flow rate was absent within the small-BPE group (rho = 0.147, p = 0.649) and lies in the direction opposite to that predicted by a fibrosis-mediated increase in urethral resistance. Stromal STAT3 correlated weakly with IPSS voiding subscore (rho = 0.261, p = 0.046, adjusted p = 0.446), voided volume (rho = 0.275, p = 0.043, adjusted p = 0.446) and post-void residual volume (rho = −0.280, p = 0.041, adjusted p = 0.446). All effect sizes were weak (∣rho∣ ≤ 0.36). We therefore treat these as chance findings and draw no inference from any of them.

The absence of correlations that survive correction is consistent with two features of the cohort. All patients were symptomatic enough to warrant surgery, so the range of symptom severity is restricted (IPSS 10–35, with 63% scoring ≥20; maximal flow rate 0.8–21 mL/s, with 95% below 15 mL/s), which attenuates correlation coefficients. In addition, IPSS and maximal flow rate did not differ between the volume groups (p = 0.695 and 0.535).

Table S3. Sensitivity analysis restricted to men aged ≥60 years: patient-level STAT3 and STAT6 mean fluorescence intensity between the small-BPE and large-BPE groups.

Compartment	Protein	Large-BPE, mean±SD (n)	Small-BPE, mean ±SD (n)	Difference (95% CI)	p	Holm p
Gland	STAT3	75.7±13.8 (43)	77.2±14.9 (13)	1.56 (−7.36 to 10.48)	0.727	1.000
Gland	STAT6	54.7±19.2 (43)	60.3±21.1 (13)	5.53 (−6.94 to 18.00)	0.378	1.000
Nodule	STAT3	142.9±45.1 (20)	140.4±27.8 (11)	−2.57 (−33.28 to 28.13)	0.865	1.000
Nodule	STAT6	57.6±14.3 (20)	58.6±14.3 (11)	0.97 (−10.02 to 11.95)	0.859	1.000
Stroma	STAT3	120.7±29.8 (42)	132.7±41.7 (14)	11.94 (−8.50 to 32.37)	0.247	1.000
Stroma	STAT6	56.6±13.7 (42)	65.5±15.2 (14)	8.87 (0.15 to 17.58)	0.046	0.277

Analysis restricted to the 57 patients aged ≥60 years (age range 62–87 years; 43 large-BPE and 14 small-BPE), excluding three patients aged 51, 53 and 54 years. Differences are small-BPE minus large-BPE (independent-samples t test); Holm adjustment across the six comparisons. For stromal STAT6, the linear mixed-effects model gave 9.69 (95% CI 0.94 to 18.44, p = 0.030) and generalised estimating equations 9.66 (95% CI 0.66 to 18.65, p = 0.035). CI, confidence interval; SD, standard deviation.

Figure S1. Patient-level distribution of STAT3 and STAT6 mean fluorescence intensity by prostate-volume group. Each point represents one patient, plotted as the mean of that patient's regions of interest within the compartment indicated. Boxes show the median and interquartile range; whiskers extend to the most extreme values within 1.5 times the interquartile range. Mean fluorescence intensity is expressed in 8-bit grey levels; all patient-level values lie within the 0–255 measurement range after correction of two data-entry errors in individual regions of interest (see Supplementary methods note). Group sizes differ between panels because not every compartment was represented in every patient's scanned fields. The *p* values shown are from independent-samples *t* tests at the patient level, uncorrected for multiple comparisons; the asterisk marks the only comparison reaching nominal significance.

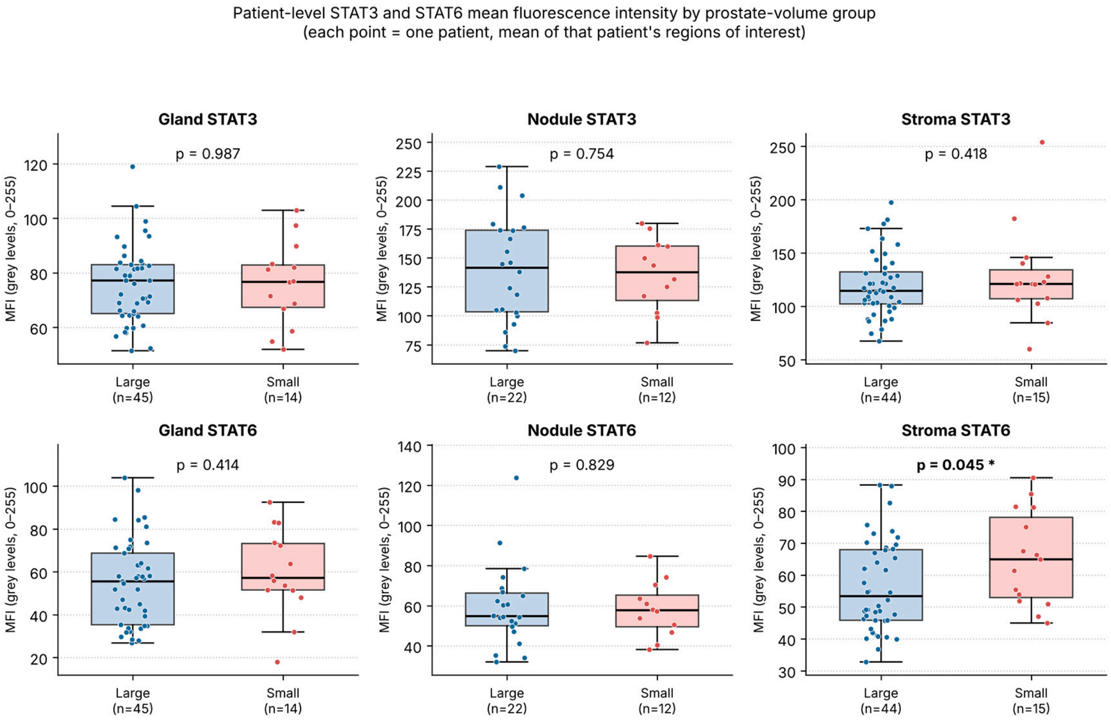

Figure S2. Negative and positive immunofluorescence controls. Immunofluorescence for DAPI (blue), STAT3 (green, Alexa Fluor 488) and STAT6 (red, Alexa Fluor 568), shown as individual channels and merged. All sections were stained in a single batch and imaged at ×200 in one session on an upright widefield epifluorescence microscope (DM2500 with DFC7000 T camera; Leica Microsystems), with each channel acquired sequentially through its filter set. (A, B) Benign prostatic tissue from a patient not included in the study cohort, imaged with identical camera settings for each channel (STAT3 and STAT6, 300 ms, gain 3; DAPI, 100 ms, gain 2): (A) negative control with the primary antibodies omitted, showing minimal signal in the STAT3 and STAT6 channels with preserved nuclear staining; (B) positive control stained with the complete protocol, showing distinct signal in both channels. (C, D) Prostate cancer tissue, corresponding regions of serial sections from the same block: (C) negative control with the primary antibodies omitted (STAT3 and STAT6, 300 ms, gain 2); (D) positive control stained with the complete protocol (STAT3, 250 ms, gain 3; STAT6, 300 ms, gain 3). DAPI was acquired at 100 ms, gain 2 in both. Because (D) was acquired with a higher

camera sensitivity than (C), panels (C) and (D) are intended for qualitative comparison only. Faint punctate signal present in the negative controls is attributable to tissue autofluorescence (lipofuscin and corpora amylacea). Brightness and contrast were not adjusted after acquisition. The controls provide qualitative support for staining specificity; matched acquisition settings do not exclude section-to-section differences in background fluorescence. Scale bars = 100 µm.

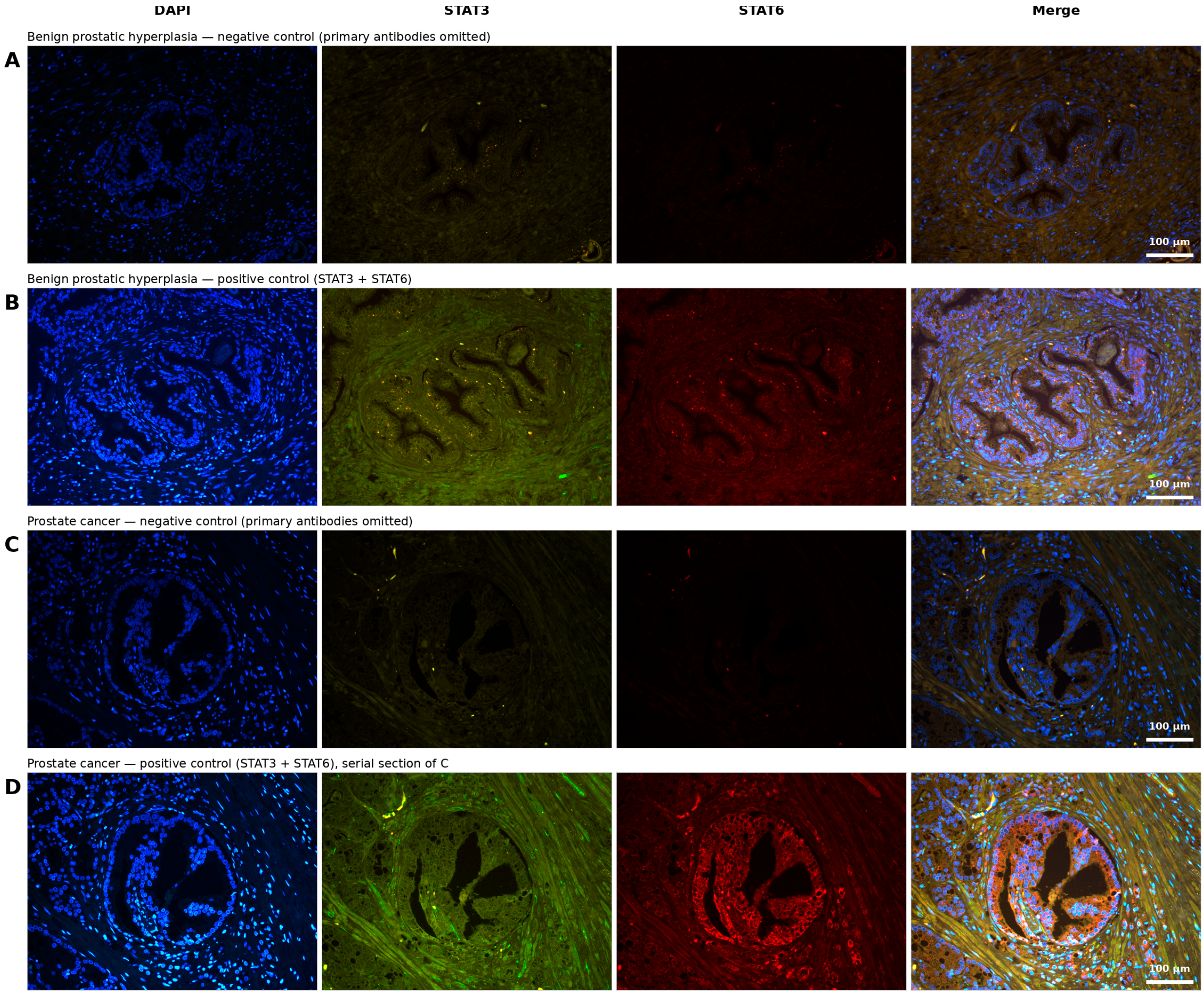

Supplementary methods note

The unit of analysis in the main text is the patient. Patient-level values were obtained by averaging the regions of interest scanned for each patient within each compartment, so that every patient contributes equally regardless of how many regions were available. Five confocal fields were acquired per patient. Within each field, glandular, stromal and nodular regions were segmented separately, and a compartment absent from a given field contributed no region. This yielded 201 glandular regions from 59 patients (median 3 per patient, range 1–5), 173 stromal regions from 59 patients (median 3, range 1–5) and 54 nodular regions from 34 patients (median 1.5, range 1–3).

Analyses were performed using SPSS version 26.0 (IBM Corp., Armonk, NY, USA) and Python 3.11 with the statsmodels package. Mixed-effects models were fitted by restricted maximum likelihood with a random intercept for patient; generalised estimating equations used an exchangeable working correlation with the patient as the cluster. Intraclass correlation coefficients were computed as the ratio of between-patient variance to total variance from the mixed model.

During revision, two regional values that exceeded the 8-bit measurement range were traced to data-entry errors and corrected against the original image-analysis output

(a nodular STAT3 value entered as 264.7 instead of 246.7 and a stromal STAT3 value entered as 259.3 instead of 253.9). The corrections changed only the STAT3 estimates slightly; no STAT6 estimate changed. The upper bound of the small-BPE group is reported as ≤33 mL, which is the largest prostate volume in that group; the threshold of <30 mL stated in the earlier version did not apply to three patients (30, 32 and 33 mL). One small-BPE patient who was unable to void during uroflowmetry (maximal flow rate and voided volume of 0) is excluded from all uroflowmetry-based variables in Table S2.
